# Differences in Prokaryotic Community Composition Between Two Climatically Contrasting Years in an Arctic Fjord Ecosystem

**DOI:** 10.1111/1758-2229.70282

**Published:** 2026-04-01

**Authors:** Eva Lopes, Miguel Semedo, Allison Bailey, Philipp Assmy, Catarina Magalhães

**Affiliations:** ^1^ Faculty of Sciences University of Porto Matosinhos Portugal; ^2^ Interdisciplinary Centre of Marine and Environmental Research (CIIMAR) University of Porto Matosinhos Portugal; ^3^ Norwegian Polar Institute Fram Centre Tromsø Norway

**Keywords:** Arctic, Kongsfjorden, phytoplankton bloom, prokaryotes, seasonality

## Abstract

Kongsfjorden, a glaciated fjord in the Arctic, is shaped by Atlantic water inflow and seasonal glacier melt. This study compared prokaryotic community dynamics during spring and summer in two contrasting years, 2019 and 2020. In spring 2019, warmer Atlantic water increased temperatures and nutrients, supporting a *Phaeocystis pouchetii*‐dominated bloom. In contrast, spring 2020 was colder, with extensive sea ice and a bloom succession from diatoms to *P. pouchetii*. Summer surface waters showed strong glacier runoff influence and low‐biomass, flagellate‐dominated phytoplankton. Greater prokaryotic diversity was observed in 2019, particularly at the surface, which was supported by network analysis revealing fewer interactions among prokaryotes in surface waters during 2020 compared to 2019. Prokaryotic community composition clearly differed between the 2 years, showing vertically homogeneous communities and higher cyanobacterial abundance in 2019, while in 2020 communities were more vertically heterogeneous and potentially locally adapted. These patterns reflect greater Atlantic water influence in 2019 versus more stratified Arctic conditions in 2020. Surface and bottom communities showed contrasting trends, shaped by depth‐specific environmental conditions. Our findings highlight the sensitivity of Arctic microbial communities to shifts in hydrography and bloom dynamics and provide important perspectives on the ecological stability and dynamics of Arctic microbial communities under changing environmental conditions.

## Graphical abstract

Seasonal dynamics of prokaryotic communities in Kongsfjorden were driven by contrasting hydrographic conditions in 2019 and 2020. Atlantification enhanced prokaryotic diversity and interactions in 2019, while sea ice and glacial runoff in 2020 reduced surface diversity and network complexity, underscoring Arctic prokaryotic sensitivity to environmental shifts.

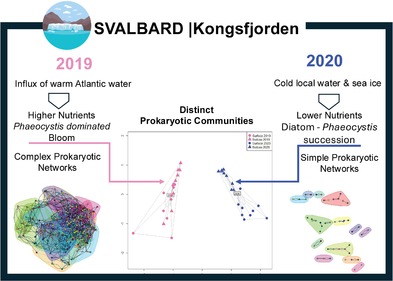

## 1 Introduction

The Arctic Ocean is one of the planet's harshest environments, characterised by extreme seasonality from polar night to 24 h of daylight and sea ice cover for large parts of the year (Meyer et al. 2017). At the same time, the Arctic is warming nearly four times faster than the global average (Rantanen et al. 2022), with the Barents Sea region, including the Svalbard archipelago, warming the fastest (Isaksen et al. 2022). Kongsfjorden is an open glacial fjord situated on the west coast of Spitsbergen, an island in the Svalbard archipelago. It has been recognised as a crucial European site for Arctic biodiversity monitoring and an excellent location for investigating potential climate change impacts in coastal ecosystems (Wlodarska‐Kowalczuk and Weslawski 2001; Hop et al. 2002; Hop and Wiencke 2019).

Physical changes in the marine environment around Svalbard are primarily driven by increased advection of warm and saline Atlantic Water (AW) with the West Spitsbergen Current (Årthun et al. 2012; Pavlov et al. 2013). Increased advection of AW from the West Spitsbergen Current is also prevalent in Kongsfjorden (de Rovere et al. 2022). This warming trend is consistent with observations in the wider Arctic Ocean, a phenomenon known as Atlantification (Polyakov et al. 2017). The increased Atlantic inflow leads to the warming of water and sea ice loss along the west coast of Svalbard and, combined with rapid atmospheric warming, has resulted in accelerated glacier melting and retreat (Li et al. 2025). As a result, the input of sediment‐rich meltwater to Svalbard fjords has increased, causing the darkening and freshening of surface waters during summer (Piquet et al. 2010; Konik et al. 2021). Consequently, the combination of warm AW, freshwater input from glaciers, and higher sediment concentrations significantly impacts the water column biogeochemistry of the region (Hodal et al. 2012; Fransson et al. 2016; Halbach et al. 2019; Nguyen et al. 2022).

These physical and biogeochemical alterations have cascading effects on biological communities. At the microbial level, Atlantification is driving notable changes in community composition, diversity, and functionality (Paulsen et al. 2016), such as altering the dynamics of phytoplankton blooms, including their timing and duration, which directly affect prokaryotic communities and the broader Arctic food web (Carmack et al. 2004; Husson et al. 2024). Warmer water temperatures can also raise microorganisms' metabolic rates, which can change the local carbon pool and ultimately boost primary productivity (Bailey et al. 2021). On a seasonal level this is reflected in increased bacterial productivity, abundance, and active cell counts during springtime (Piquet et al. 2015) and a shift in dominance of cold‐adapted bacteria, such as *Polaribacter* (Bacteroidota) and *Colwellia* (Proteobacteria), in winter towards temperate‐associated taxa like *Pseudomonas* and *Alteromonas* (Proteobacteria) later in the season (Wietz et al. 2021). In Kongsfjorden, reduced sea ice formation since 2006 has led to earlier spring phytoplankton blooms, altering the timing, composition, and availability of organic matter to other organisms (Hegseth et al. 2019; Assmy et al. 2023; Hoppe et al. 2024). The seasonal transition of prokaryotic communities is closely linked to the dynamics of phytoplankton. During early blooms, specific prokaryotic taxa, such as Alphaproteobacteria (Proteobacteria), dominate, whereas later in the season, distinct groups, including Flavobacteriia (Bacteroidota) and Gammaproteobacteria (Proteobacteria), become more prevalent (Piquet et al. 2010; Cardman et al. 2014). Chemolithoautotrophic marine Crenarchaeota disappear from surface waters in late spring and summer, underscoring the linkage between microbial and phytoplankton dynamics. Water depth also influences phytoplankton and prokaryotic community composition, with microbial diversity generally increasing with depth in Kongsfjorden (Zeng et al. 2013; Jain et al. 2019). Freshwater prokaryotes introduced via meltwater rivers can also imprint on microbial diversity in glaciated fjords (Kachiprath et al. 2024).

These changes highlight the sensitivity of Arctic Ocean microbial ecosystems to climate‐induced changes in the environment, underscoring the need for long‐term monitoring and predictive modelling to understand future ecological shifts across Svalbard fjord systems. Earlier Arctic microbial studies were limited by infrequent sampling, resulting in a limited understanding of rapid and transitional changes in microbial communities (e.g., Sinha et al. 2017). Recent works, such as Liu et al. (2025), have begun to use network analysis to infer interactions among the communities, but typically at broad spatial scales. This study addresses those limitations by employing high‐frequency, weekly seasonal sampling during the spring and summer seasons of 2019 and 2020 as part of the Kongsfjorden long‐term seasonal pelagic monitoring programme, and by pairing it with detailed network analysis, linking short‐term community shifts and potential biotic interactions throughout key seasonal transitions in the Arctic productive season.

Here, we present the results of the first 2 years of the Kongsfjorden long‐term seasonal pelagic monitoring programme (Assmy et al. 2023), including seawater characteristics and prokaryotic community structure from spring until late summer. This study aimed to assess the weekly fluctuations of prokaryotic communities in Kongsfjorden during the spring and summer seasons of two climatically contrasting years, characterised by differences in water masses, physicochemical parameters, and glacier freshwater inputs. By elucidating these dynamics, this research not only enhances our fundamental knowledge of microbial ecology in one of the planet's most rapidly changing regions but also contributes to broader efforts in predicting the future impacts of climate change on marine biodiversity and biogeochemical cycles.

## 2 Experimental Procedures

### 2.1 Study Area

**FIGURE 1:**
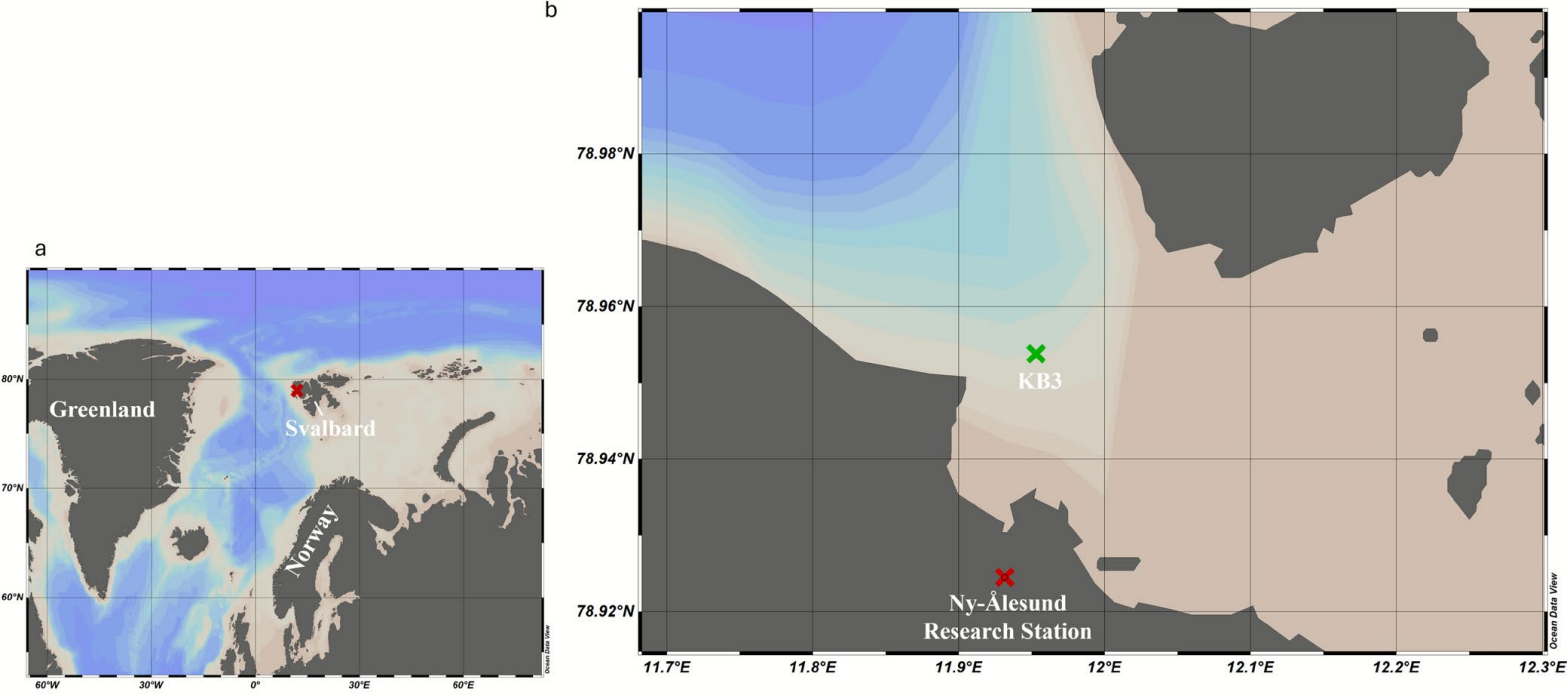
Study area location. (a) Map showing the regional context, including Svalbard (Norway), with the Ny‐Ålesund Research Station marked by a red X. (b) KB3 station, marked by a green X, indicating the site where samples for this study were collected. The red X represents the location of the Ny‐Ålesund Marine Lab, where the samples were analysed and stored.

This study was conducted in Kongsfjorden (Figure 1a), a glaciated fjord situated on the northwest coast of Spitsbergen of the Svalbard Archipelago, Norway (79 ° N, 11–12 ° E). This open fjord (no distinct sill at its entrance) is strongly influenced by the advection of warm Atlantic Water from the West Spitsbergen Current (Hop et al. 2002; Svendsen et al. 2002; Cottier et al. 2005; Hop et al. 2006). Kongsfjorden water masses are a mixture of warm, saline Atlantic Water and colder, less dense fjord waters. The fjord exhibits seasonal stratification, with stronger vertical density gradients in summer due to freshwater inputs and more homogeneous conditions in winter (Cottier et al. 2005; Tverberg et al. 2019). These characteristics make this area an ideal location to analyse the influence of Arctic environmental gradients on the dynamics of planktonic prokaryotic communities.

### 2.2 Sample Collection

The samples in this study were obtained from mid‐fjord station KB3 (78°57 N, 11°57 E), using the vessel MS Teisten (Kings Bay AS, Ny‐Ålesund). Sampling began in late April (2019) and early May (2020) and finished in early September for both years (Table 1), covering the spring and summer seasons. The KB3 station presents a depth range of 340 m, and seawater samples for prokaryotic community analysis were collected at two depth layers: Surface, 15 m, and Bottom, 300 m, using a 10 L Niskin bottle (Ocean Test Equipment Inc., Fort Lauderdale, FL, USA). On July 28, 2020, additional samples were collected aboard RV Kronprins Haakon at depths of 25 and 339 m (Table 1). Forty samples, in total, were collected during the two years (2019: *n* = 20, 2020: *n* = 20). For the prokaryotic community analysis, seawater samples were filtered through a Sterivex filter (0.22 μm pore size; SVGV010RS; Merck Millipore) with a peristaltic pump. The filtered volume of the samples varied from 1.2 to 5 L, with lower volumes resulting from occasional filter clogging that prevented completion of the full 5 L. The filters were sealed and stored at −80°C. Then, the filters were transported in dry ice to CIIMAR for later DNA extraction.

**TABLE 1:** Sampling information regarding dates and depth corresponding to the prokaryotic communities' sampling. Water samples were collected consistently across the sampling season; only those with sufficient sequencing quality were retained for downstream analysis.

Year	Date	Depth (m)
2019	29 April	300
	07 May	15 and 300
	13 May	15 and 300
	20 May	15 and 300
	27 May	15
	10 June	15 and 300
	20 June	300
	03 July	300
	09 July	15
	15 July	15
	22 July	15 and 300
	29 July	300
	06 August	15
	26 August	15
	02 September	15
2020	08 May	15 and 300
	12 May	15 and 300
	19 May	15 and 300
	25 May	15 and 300
	04 June	15 and 300
	09 June	15 and 300
	17 June	15 and 300
	28 July	25 and 339
	02 September	15 and 300
	08 September	15 and 300

### 2.3 Environmental Parameters

At KB3, salinity, temperature (°C), chlorophyll fluorescence (μg/L), and turbidity (Formazin Turbidity Units, FTU) were measured using an STD profiler (MiniSTD model SD‐204, SAIV A/S, Bergen, Norway). Samples were collected for chlorophyll‐*a* at 15 m, while nutrient concentrations (ammonium, nitrite, nitrate, silicic acid, and phosphate) were measured at both 15 and 300 m. Nutrient samples were filtered on‐board immediately after sample collection with 0.2 μm syringe filters (Whatman SFCA, UK) and syringes pre‐rinsed with the sample. The filtered water was then sealed and stored at −20°C. Prior to analysis, the samples were thawed for 24 h. Colourimetric analysis was performed on a QuaAAtro autoanalyser (Seal Analytical, Mequon, USA), utilising internal calibrations and Certified Reference Materials (KANSO, Osaka, Japan) for quality control. Regarding the chlorophyll‐*a* samples, water from each depth was transferred directly into 1 L brown plastic bottles and kept cool while brought to the Marine lab in Ny‐Ålesund (Figure 1b) for filtering. Chlorophyll‐*a* samples were filtered through GF/F filters (Whatman, Maidstone, UK) and then extracted directly in 5 mL methanol for 12 h at 4°C in the dark (Holm‐Hansen and Riemann 1978). Chlorophyll‐*a* and Phaeopigments concentrations in extracts were measured on a Trilogy Laboratory fluorometer (Turner Designs, CA, USA) before and after acidification, respectively, with two drops of 5% HCl (Holm‐Hansen and Riemann 1978). A detailed description of chlorophyll‐*a* and nutrient methodologies can be found in Assmy et al. (2023).

### 2.4 DNA Extraction and 16S rRNA Gene Sequencing

DNA extraction from the Sterivex filters was performed using the PowerWater Sterivex DNA Isolation Kit protocol (5091 4600‐50‐NF; Qiagen), following the respective manufacturer's instructions. The extracted DNA was quantified using the Qubit dsDNA High Sensitivity kit (Q32851; Invitrogen). The 16S rRNA gene was amplified with the degenerate primer pair 515YF (5′‐GTGYCAGCMGCCGCGGTAA–3′) and Y926R‐jed (5′–CCGYCAATTYMTTTRAGTTT–3′), targeting the hypervariable region V4–V5 (Caporaso et al. 2011; Apprill et al. 2015; Parada et al. 2016). PCR reactions were done according to the following protocol: 3‐min denaturation step of 95°C, followed by 25 cycles of 98°C for 20 s, 60°C for 30 s, and 72°C for 30 s, and finally, an extension stage at 72°C for 5 min. The amplified products produced the libraries for the Illumina paired‐end 2 × 250bp sequencing on an Illumina MiSeq platform, using V3 Chemistry (Illumina). Sequencing was performed by the Integrated Microbiome Resource (IMR), following their protocol (https://imr.bio/protocols.html).

### 2.5 Bioinformatic Analysis

The raw FASTQ files obtained with Illumina MiSeq were trimmed for primer removal using “cutadapt” v.1.16 and imported into R (version 4.1.2) using “DADA2” package v.1.28.0 (Callahan et al. 2016). Sample filtering, trimming, error rates learning, dereplication, and amplicon sequence variant (ASV) inference were performed with default settings. Chimaeras were removed with the *removeBimeraDenovo* function using the method “consensus”. The taxonomy analysis was performed using the phyloseq R package (v. 1.38.0; McMurdie and Holmes 2013), with taxonomy assigned through the native implementation of the naive Bayesian classifier and a DADA2‐formatted reference database derived from the SILVA v138.1 database for 16S taxonomical analysis (Quast et al. 2013). The pre‐processing steps resulted in 3642 ASVs, with a median number of 39,574 reads per sample (Supplementary Table 1; Supplementary Figure S1). Taxonomy filtering was performed by removing eukaryotic, mitochondrial, and chloroplast sequences from the 16S database. Relative abundances of each ASV per sample were calculated in the filtered dataset by dividing the absolute abundance (counts) of each ASV by the sum of counts of all ASVs in each sample.

To estimate species richness and α‐diversity of the prokaryotic communities, the number of observed ASVs and the Shannon index were calculated. The Shannon index accounts for both abundance and evenness of the species (Shannon 1948). Before β‐diversity analyses, abundance data were log‐transformed using the vegan package (v. 2.6–4; Oksanen et al. 2020), following Anderson et al. (2006). In this transformation, zeros were left as zeros. Temperature–salinity (TS) diagrams were generated using the gg Ocean Plots R package (v. 0.1.6; Vihtakari 2021) to visualise and classify water masses sampled during the study.

### 2.6 Statistics and Reproducibility

The within‐group distance of the centroids was calculated to represent the β‐diversity of the prokaryotic communities based on dissimilarity among samples, using the vegan R package (v. 2.6–4; Oksanen et al. 2020). A Canonical Correlation Analysis (CCA) and an envfit test, which assesses the significance of environmental variables in explaining the dissimilarity between samples, were also performed to determine whether the different environmental parameters, including the abundance of diatoms and *Phaeocystis* (previously analysed in Assmy et al. (2023)), correlated and influenced the observed dissimilarities. Significant relationships were considered at *α* = 0.05. A network analysis was performed to visualise and evaluate co‐occurrence patterns between prokaryotic ASVs, using the Sparse InversE Covariance estimation for Ecological Associations and Statistical Inference (SPIEC‐EASI) package (Version 1.1.3; Kurtz et al. 2015). To avoid the effect of weak and autocorrelations and samples with limited sequencing depth, the dataset was subjected to a rigorous subsampling process. Initially, samples with fewer than 5000 reads were removed. Then, ASVs with zero counts in this sample subset were deleted. This was followed by rarefying all samples to the minimum read count of 5445 reads. After this initial data filtering, four separate subsets were created: Surface 2019, Bottom 2019, Surface 2020, and Bottom 2020. Finally, low‐abundance ASVs (that do not appear more than 1 time in at least 3 samples of each subset) were excluded. The networks were run using Meinshausen Buhlmann's (MB) neighbourhood selection method and an optimal sparsity threshold of 0.05 (Liu et al. 2010). For the network visualisation, a plot was made with the igraph R package (v. 1.4.2; Csardi and Nepusz 2006), and subcommunities/modules were found using the Louvain method (Blondel and Guillaume 2008).

All data analyses were performed using RStudio (version 4.1.2, Copyright 2021, the R Foundation for Statistical Computing). The majority of plots were obtained with base R and the ggplot2 R package (v. 3.5.0; Wickham 2016).

## 3 Results

### 3.1 Environmental Differences Between the Two Years

**FIGURE 2:**
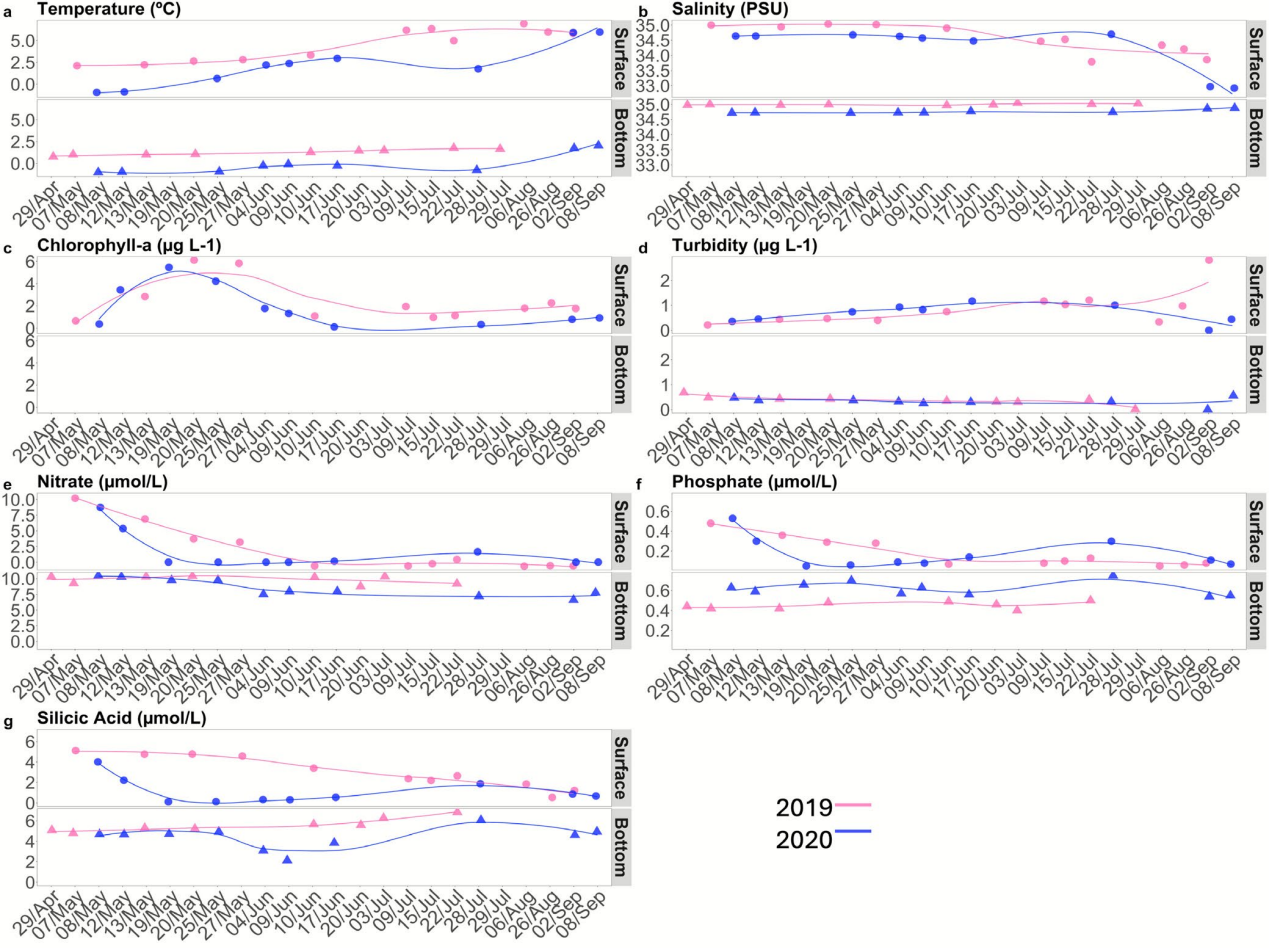
Environmental and biogeochemical properties (a: Temperature; b: Salinity, c: Chlorophlyll‐a; d: Phaeopigments; e: Nitrate; f: Phosphate; g: Silicic Acid) of the years 2019 (blue) and 2020 (blue).

Surface and bottom ocean temperatures were higher in 2019 compared to 2020 and showed gradual warming from spring to summer in both years, which was most pronounced in surface waters (Figure 2a). The surface water temperature in 2019 varied from 2.12°C to 6.95°C and from −0.95°C to 5.92°C in 2020. The water temperature at the bottom varied from 0.77°C to 1.77°C in 2019 and from −1.02°C to 1.75°C in 2020. Salinity followed a similar pattern, with generally higher salinities in 2019 compared to 2020 in both surface and bottom waters (Figure 2b). This demonstrates the prevalence of Atlantic water masses during the early spring and summer of 2019, especially in the spring (Supplementary Figure S2). In the spring of 2020, colder local water masses were prevalent (Assmy et al. 2023; Supplementary Figure S2). Vertical differences in salinity over time were also observed. While bottom salinities stayed relatively stable throughout the observational period, surface salinities declined in June 2019 and September 2020, respectively. Chlorophyll‐*a* in surface waters (Figure 2c) peaked in May for both years, then decreased until early July, and increased again towards the end of the sampling season (late July—early September). The pronounced chlorophyll‐*a* increase in May marked the phytoplankton spring bloom in both years. Surface turbidity showed a gradual seasonal increase from spring to mid‐July in both years and diverging patterns thereafter, with a steep increase in late August 2019 and a decline in early September 2020 (Figure 2d). Turbidity at the bottom depth was generally lower than in surface waters and stayed relatively constant throughout the sampling period for both years. Surface nitrate concentrations showed a decline in spring, which was more pronounced in 2020 from 8.7 to 0.01 μM L^−1^ compared to 10.2 to 3.2 μM L^−1^ in 2019 (Figure 2e). Bottom nitrate concentrations remained high and relatively stable throughout the summer, with a slight decrease in 2020. Phosphate demonstrated similar patterns to nitrate across the water column and between the years (Figure 2f). Surface silicic acid concentrations showed a similar steep decline in spring 2020 as nitrate and phosphate, while they remained high and relatively stable until late May 2019 and showed a more gradual decline thereafter (Figure 2g). Bottom silicic acid concentrations in 2019 showed a gradual increase over time, while they were more variable in 2020.

### 3.2 Prokaryotic Diversity and Environmental Relationships

**FIGURE 3:**
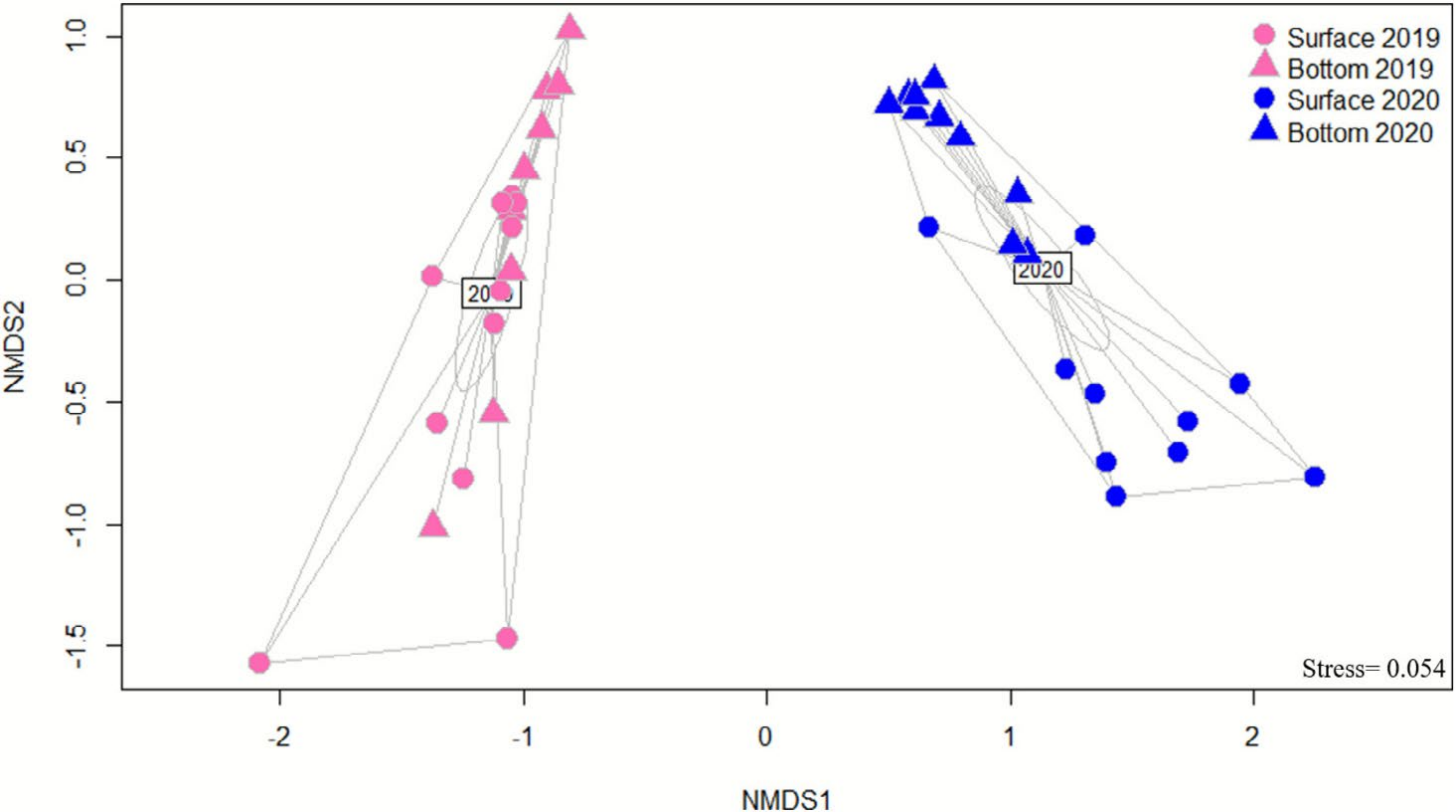
Bray–Curtis dissimilarity of the prokaryotic communities between the years 2019 and 2020 (Stress = 0.054).

To understand differences in the prokaryotic community structure, a beta diversity analysis was performed using Bray–Curtis Dissimilarity. This analysis revealed that prokaryotic communities were mostly separated by year rather than by sampling depths (Figure 3). All samples collected in 2019 can be found in a cluster different from those in 2020 in the nMDS (Non‐metric Multidimensional Scaling) analysis. Samples from 2019 were relatively heterogeneous and did not show any specific depth pattern, while samples from 2020 showed distinct groupings for surface and bottom waters. This pattern was statistically validated by a permutational multivariate analysis of variance (PERMANOVA), where the interaction between year and depth was significant (*p* < 0.05; Supplementary Table S2). The greater heterogeneity of 2019 samples and the more distinct depth‐related groupings in 2020 are further supported by a quantitative analysis of centroid distances (Supplementary Figure S3). To assess variance homogeneity, a beta dispersion analysis was performed, revealing significant differences in dispersion across depths (*p* = 0.008; Supplementary Table S2), but not between the sampling year (*p* = 0.395; Supplementary Table S2), indicating that community variability was more strongly influenced by vertical stratification than by temporal factors.

**FIGURE 4:**
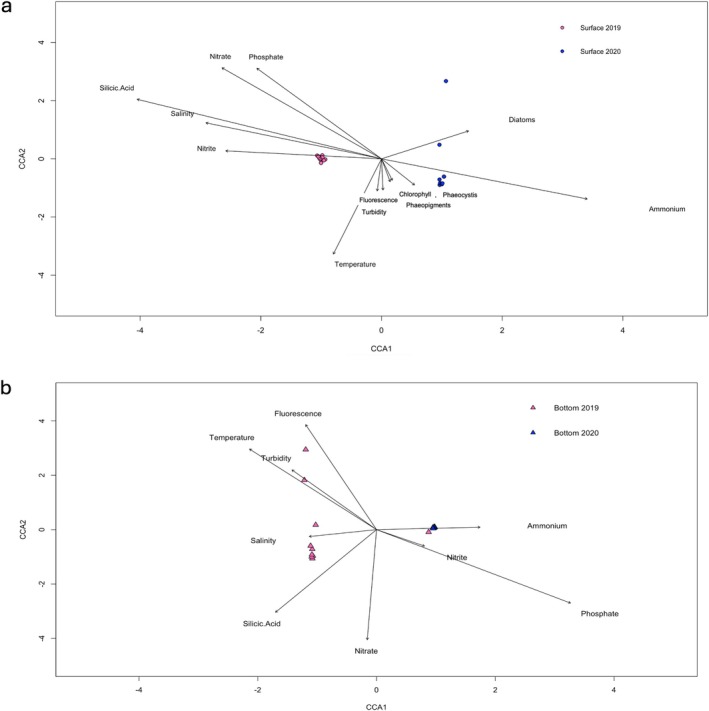
Canonical Correlation Analysis (CCA) showing the influence of environmental variables on the distribution of prokaryotic community composition of the different depths (Surface‐ a; Bottom‐ b).

A Canonical Correlation Analysis (CCA) and an Envfit test for each depth were performed to examine the relationships between prokaryotic community structure, physicochemical parameters, and the abundance of diatoms and *Phaeocystis* (Figure 4, Supplementary Table S3). Both depths showed significant relationships with nitrate, phosphate, and silicic acid (Supplementary Table S3). Temperature and chlorophyll fluorescence were also statistically significant with the bottom communities (Supplementary Table S3). The communities collected at the surface in 2020 were more heterogeneous and more associated with the presence of diatoms (Figure 4a). At the bottom, samples from 2019 proved to be more heterogeneous and exhibited a stronger association with the physicochemical parameters (Figure 4b). In both surface and bottom CCA analyses, the second canonical axis (CCA2) appeared to reflect seasonal progression: for surface samples (Figure 4a), the positive end of CCA2 corresponded to the earliest samples collected in 2020, while for bottom samples (Figure 4b), the positive end aligned with the latest samples collected in 2019.

**FIGURE 5:**
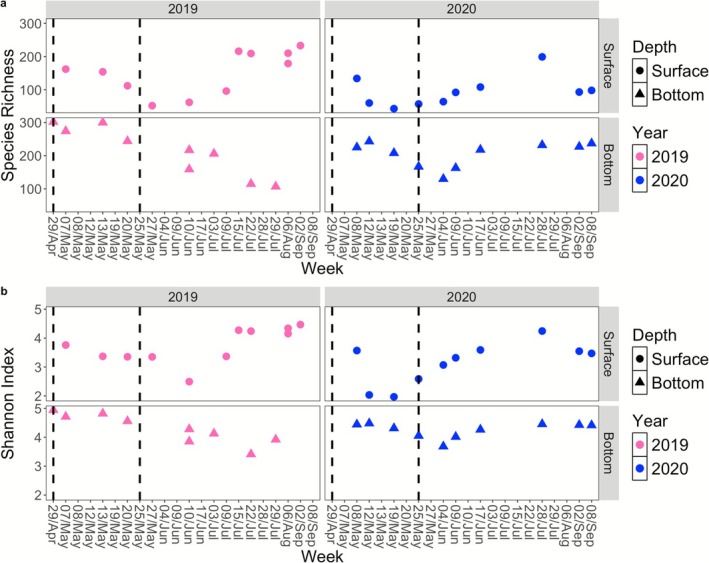
Alpha diversity measures (a: Species Richness; b: Shannon Index) at the KB3 station between the years 2019 and 2020 for two different depths (Surface and Bottom). Dashed lines correspond to the spring bloom period (late April to May).

Both sampling periods exhibited similar patterns in species richness and diversity (Figure 5), confirmed in the rarefied dataset (Supplementary Figure S4). At the surface, species richness decreased during the spring bloom, from late April to May in both years, reaching its lowest point in late May (Figure 5a). It then began to increase from June onward. However, a significant decrease was observed in September 2020. In contrast, bottom samples in 2019 showed the opposite trend. Species richness was high during the spring bloom but declined afterward. In 2020, bottom samples also had relatively high richness during the bloom but decreased as they progressed, then increased again in June, peaking on June 17 (Figure 5a). The Shannon Index at the bottom followed a similar trend (Figure 5b). It peaked during the spring bloom in 2019 and gradually declined throughout the summer. In 2020, bottom samples showed high Shannon Index values during the bloom, followed by a decline and a recovery in June, forming a V‐shape pattern (Figure 5b). However, surface samples displayed an inverse Shannon Index pattern compared to the bottom samples. After the spring bloom, the Shannon Index started to increase, reaching its peak in July in both years.

To assess the relationship between bloom indicators and species diversity metrics, a linear regression was conducted using chlorophyll fluorescence and chlorophyll‐*a* and the selected diversity metrics. Chlorophyll fluorescence showed a statistically significant relationship with both species richness and diversity (Linear regression, *p* < 0.05; Supplementary Table S4).

### 3.3 Community Composition and Temporal Variability

**FIGURE 6:**
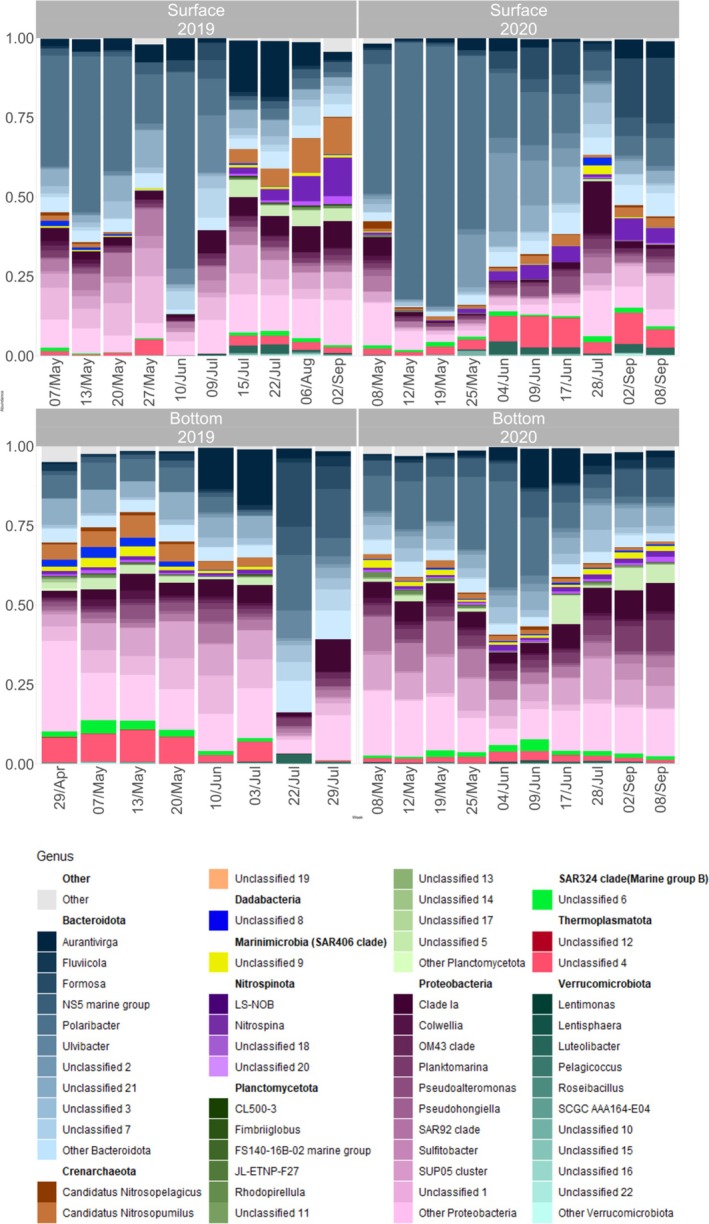
Relative abundances of the top 10 phyla and their genera.

Prokaryotic community composition revealed differences across depths and years. In Figure 6, it is possible to observe the top 10 phyla distribution and their genera between spring and summer of 2019 and 2020.

The top 10 phyla of both years displayed pronounced seasonal dynamics in surface waters (Figure 6). Bacteroidota and Proteobacteria dominated throughout the sampling period in both years, particularly during the spring bloom, and with a few exceptions, Bacteroidota showed a higher relative abundance in samples from 2020 (Figure 6). In this study, phyla considered less common based on their overall lower relative abundances exhibited higher relative abundances in 2019 compared to 2020 (Supplementary Table S5). For example, cyanobacteria were less abundant in 2020 compared to 2019 (Figure 6). In the post‐bloom phase, prokaryotic diversity increased in surface waters (Figure 5) with phyla such as Crenarchaeota and Nitrospinota appearing, particularly evident in July and early September 2019 (Figure 6). Bottom waters exhibited greater stability in prokaryote community composition across seasons and years compared to the surface, except in late July 2019 (Figure 6). However, small differences in bottom waters were observed, such as the higher abundance of Dadabacteria during the 2019 spring bloom relative to 2020 (Supplementary Table S5). Although bottom waters showed little seasonal variation overall, subtle shifts were noted. For instance, the emergence of genera such as *LS‐NOB* towards the end of 2020 was a notable change (Figure 6). Both taxonomic composition (Figure 6) and alpha diversity (Figure 5) suggested vertical mixing between surface and bottom waters. For instance, Crenarchaeota and Planctomycetota were abundant at the bottom during late spring and early summer of 2019. They later became dominant in surface waters after the 2019 bloom, coinciding with an increase in surface alpha diversity (Figure 5).

The vertical distribution of prokaryotes revealed interesting patterns of community dynamics. The observed increase in Crenarchaeota in surface waters during the post‐bloom phase in 2019 was primarily driven by an increase in the relative abundance of the genus Candidatus Nitrosopumilus, which belongs to this phylum. This transition in surface waters was accompanied by substantial increases in diversity and species richness, contrasting with the relatively stable, low‐diversity communities observed during the bloom period (Figure 5). During the bloom period, nitrate concentration (Figure 2e) was high in surface waters, whereas the presence of *Candidatus Nitrosopumilus* was low. After the bloom, the abundance of *Candidatus Nitrosopumilus* began to increase at the surface, and the nitrate concentration started to decrease significantly (Figure 2e). Similar trends were evident in 2020, although less pronounced, with the emergence of phyla such as Thermoplasmatota and Planctomycota contributing to post‐bloom community shifts starting in June.

### 3.4 Interactions Within the Prokaryotic Community

**FIGURE 7:**
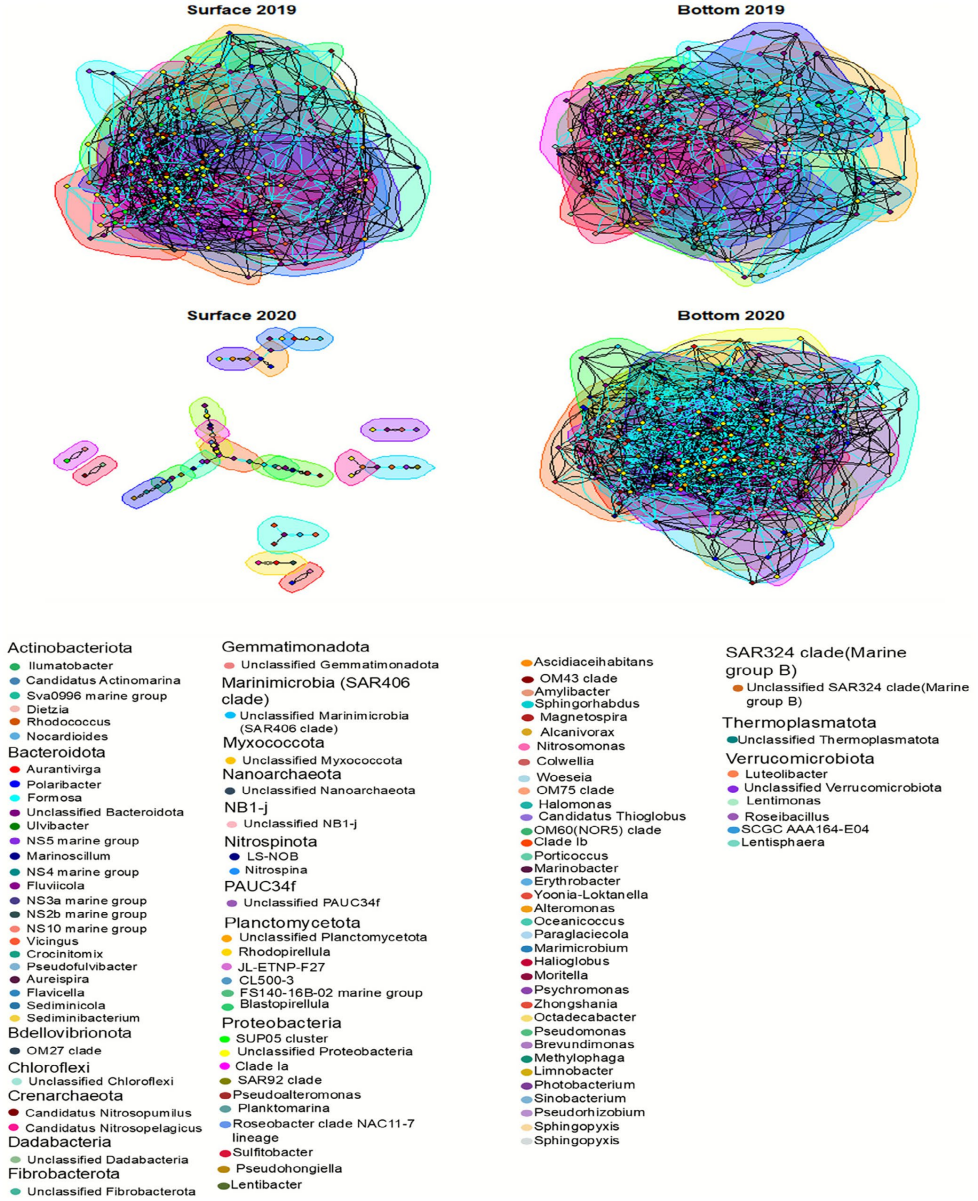
Network analysis of the overall communities of the four different sets. The network was inferred using SPIEC‐EASI with the Meinshausen‐Bühlmann (MB) neighbourhood selection method. Edges represent significant co‐occurrence or exclusion interactions, determined by stability selection with a threshold of 0.05. Cyan lines indicate negative associations, while black lines indicate positive associations.

Co‐occurrence networks were constructed for each year and depth layer (Figure 7).

In total, 3544 interactions (edges) were found in the four networks generated (Table 2). Most of the interactions were positive (62.87% on average). Within each network, it was observed that ASVs, represented as vertices, often had a degree (number of edges linked to vertices) higher than three, suggesting that many taxa are involved in multiple potential interactions (Supplementary Figure S5). A substantial difference was observed between the interaction networks of the two study periods. While in 2019, the number of interactions did not vary much across the water column (Surface = 930, Bottom = 832), in 2020, there was a drastic difference in these interactions (Surface = 99, Bottom = 1683), with the surface prokaryotic community of 2020 showing a minimum number of significant interactions compared to all other networks (Table 2). By employing the Louvain cluster method, several modules were found (Surface 2019 = 19; Bottom 2019 = 17; Surface 2020 = 21; Bottom 2020 = 17). The surface of the year 2020 exhibited the strongest structural integrity, characterised by high modularity (Modularity = 0.90). This structure reflects strong internal correlations among vertices.

**TABLE 2:** Output of network analysis.

	Surface 2019	Bottom 2019	Surface 2020	Bottom 2020
Number of Samples	10	9	10	10
Number Vertices	251	239	76	319
Number Edges	930	832	99	1683
Number Modules	19	17	21	17
Modularity	0.61	0.63	0.90	0.52
Positive Interactions	642	545	58	983
Positive %	69.03	65.5	58.59	58.41
Negative Interactions	288	287	41	700
Negative %	30.97	34.5	41.41	41.59
Exclusive Genera	9	1	1	13

Even though the number of interactions at the surface in 2020 was lower compared to the other datasets, it was the only dataset that contained taxonomically exclusive modules, which were modules in which all genera belonged to the same phylum. Specifically, certain genera were only found within the 2020 surface network modules. One module consisted of genera belonging to the phylum Bacteroidota (Module 3: Polaribacter and unclassified genera), while another included genera from the phylum Proteobacteria (Module 16: Pseudohongiella, Ascidiaceihabitans, and unclassified genera). Among the different interactions found in the various networks, it was possible to find some exclusive ones. Unclassified genera of the phyla Nanoarchaeota and Myxococcota, *Halioglobus*, *Marinobacter*, *Paraglaciecola*, *Marimicrobium* (Proteobacteria), *Pseudofulvibacter* (Bacteroidota), *Ilumatobacter* (Actinobacteriota), and *Roseibacillus* (Verrucomicrobiota) only exhibited significant interactions at the surface in 2019. Similarly, the genus *Psychromonas* (Proteobacteria) only showed significant interactions in the network at the bottom in 2019. In 2020, the surface network also featured one exclusive genus: *Octadecabacter*, belonging to the phylum Proteobacteria. The bottom 2020 network featured some exclusive genera (Table 2), including *Nocardioides*, *Dietzia*, *Rhodococcus* (Actinobacteriota), *Methylophaga*, *Pseudorhizobium*, *Limnobacter*, *Photobacterium*, *Idiomarina*, *Sinobacterium* (Proteobacteria), *Sediminibacterium* (Bacteroidota), *Lentisphaera* (Verrucomicrobiota), and unclassified genera from the phyla PAUC34f and Fibrobacterota.

## 4 Discussion

### 4.1 Environmental Drivers, Bloom Variability, and Prokaryotic Dynamics

Increased shelf‐fjord exchange allows warm Atlantic Water to penetrate Kongsfjorden, as observed in 2019 (de Rovere et al. 2022). This process, known as Atlantification, could lead to shifts in prokaryotic community composition, as temperature is a key factor affecting prokaryotes, particularly in low‐temperature environments (Winter et al. 2008; Sunagawa et al. 2015). Atlantic microorganisms are better adapted to warmer waters, potentially outcompeting native Arctic microorganisms (Priest et al. 2023; Hörstmann et al. 2024), which will be further enhanced by the intensifying Atlantification (Seebens et al. 2018; Frost et al. 2019; Polyakov et al. 2025). For example, cyanobacteria are known indicators of Atlantic water in the Arctic Ocean (Paulsen et al. 2016), and their higher occurrence in 2019 (Supplementary Figure S6), the year with the largest Atlantic influence in this study, may be influenced by the strength of Atlantic waters. The introduction of new microorganisms leads to changes in community diversity. Consistent with this, diversity was higher in the warm spring of 2019 than in the cold spring of 2020.

Sea ice in Kongsfjorden is usually limited to the inner fjord, but in spring 2020, its extent was unusually large compared to the past decade (Assmy et al. 2023). Glacial runoff during summer contributes to higher turbidity and lower salinity in surface waters (Cao et al. 2020; Assmy et al. 2023). In this study, surface salinity decreased, and turbidity increased during the summer in both 2019 and 2020, with these changes being more pronounced in 2020 (Figure 2b,d). Another factor influencing the communities is nutrient concentration. Cao et al. (2020) reported that the nitrate concentration increased with the advection of Atlantic water masses. This is supported by our study, which showed higher nutrient concentrations at both depths in the more Atlantic‐influenced year 2019 (Figure 2e). With these environmental changes, more pronounced at surface waters in 2019, it was possible to detect alterations in the prokaryotic communities (Figure 6). With the salinity and nitrate concentration drop and the turbidity increase, it was possible to observe the appearance of phyla such as Nitrospinota and Planctomycetota.

Beta‐diversity analysis showed significant changes in prokaryotic diversity over the years, with 2019 samples, regardless of the depth, clustering together and apart from the samples of 2020 (Figure 3). Given that the fjord in spring 2020 was dominated by local Arctic water masses (Assmy et al. 2023; Supplementary Figure S2), it is possible that this constituted a barrier for Atlantic Water and associated microorganisms to enter Kongsfjorden. This could explain why samples were clustered by year and not by depth when depth is a common and dominant driving factor in shaping microplankton community structure (e.g., Wietz et al. 2024). Therefore, despite the general expectation of depth‐related structuring, differences in water masses between the years played a dominant role in shaping community composition in this study.

Besides the differences observed between spring and summer of 2019 and 2020, variations in community composition and diversity were also evident throughout the spring bloom periods. In both sampling seasons, surface diversity and richness decreased from the start of the sampling period, reaching their lowest point during the spring bloom (Figure 5) before increasing again. This observation contrasts with a previous study that reported a general decrease in prokaryotic richness between the late spring and the summer post‐bloom (Wilson et al. 2017). The bloom diversity minimum observed in our study in surface waters is likely due to the strong dominance of a few taxa that efficiently utilise the influx of carbon and nutrients (Buchan et al. 2014). In contrast, at the bottom, diversity peaked early in the season and gradually declined. By late summer, surface prokaryote richness was significantly higher, as confirmed by linear regression against bloom indicators: richness was negatively correlated with chlorophyll fluorescence and chlorophyll‐*a* (Supplementary Table S4). This pattern is likely due to the more complex composition of dissolved organic carbon in deeper, darker waters, which are not dominated by a few bloom‐associated taxa (Alonso‐Sáez et al. 2008).

Prokaryotes exhibit temporal variations in abundance and composition, closely tied to phytoplankton dynamics (Piquet et al. 2015; Sinha et al. 2018). In 2019, in our study area, the inflow of Transformed Atlantic water, indicating advection from the West Spitsbergen Current, supported a *Phaeocystis*‐dominated bloom. Although *Phaeocystis* co‐dominated the spring bloom in 2020, the local polar water masses supported a spring bloom initially dominated by diatoms (Assmy et al. 2023). Piquet et al. (2015) suggested that phytoplankton blooms, particularly of diatoms and *Phaeocystis*, may trigger increased activity in Bacteroidota and Proteobacteria. The dominant phylum in this study was Bacteroidota, followed by Proteobacteria. Bacteroidota have a selective advantage when growing on complex organic matter produced by marine phytoplankton (Passow 2002; Fernández‐Gómez et al. 2013; Williams et al. 2013), resulting in a close association between this phylum and phytoplankton in cold polar waters (Grossart et al. 2005; Williams et al. 2013). Its abundance tends to increase towards the late blooms (Alonso‐Sáez et al. 2008). Even though the abundance of this phylum in this study was generally elevated, it was possible to observe an increase when the phytoplankton abundance started to decrease, especially in 2020 from May 25 to June 17 (Figure 6). Another explanation for the high presence of Bacteroidota in the water column is their “release” by sea ice melting or because of growth on organic matter liberated by thawing sea ice (Piquet et al. 2011; Bowman et al. 2012). Furthermore, the formation of extensive sea ice in 2020 led to deep convective mixing of the water column, which likely resuspended diatom resting spores. This, in turn, influenced the microbial community by altering the types and quantities of organic material available for prokaryotic consumption, contributing to a higher diatom inoculum in the 2020 spring bloom compared to 2019 (Saenko et al. 2002; Assmy et al. 2023).

Other prokaryotic groups exhibited temporal shifts during the spring bloom periods. At the surface, Crenarchaeota abundance, especially *Candidatus Nitrosopumilus*, an ammonia‐oxidising archaea, remained low early in the bloom but increased towards the end of the study period, especially in 2019 (Figure 6). These archaea play a central role in the nitrogen cycle by converting ammonium to nitrite, a key step in nitrification (Wuchter et al. 2006; Galand et al. 2009; Santoro et al. 2015). Their activity is typically suppressed during active phytoplankton blooms due to competition for ammonium (Alonso‐Sáez et al. 2008; Smith et al. 2014), but post‐bloom conditions favour their proliferation. Despite their increase, surface nitrate concentrations did not rise as expected. Instead, an inverse pattern was observed relative to *Candidatus Nitrosopumilus* abundance (Figure 2g and Figure 6), a pattern also reported in other oceanic regions (Semedo et al. 2021). In 2019, this genus appeared at the surface from July 15 (Figure 6), coinciding with a nitrate peak, while at the bottom, both Crenarchaeota abundance and nitrate levels declined after July 3 (Figure 2g and Figure 6). Additionally, carbon‐degrading Verrucomicrobia, such as *Lentimonas*, appeared towards the end of the bloom (Cardman et al. 2014; Sichert et al. 2020), further reflecting post‐bloom microbial succession.

Dadabacteria can adapt to extreme conditions during winter in the Arctic Ocean (Wietz et al. 2021) and possibly contribute to breaking down components of dissolved organic matter (Graham and Tully 2021). In this study, these prokaryotes could play a role in decomposing organic matter, given that they were mainly found during the bloom period, especially at the bottom of 2019 (Figure 6). Verrucomicrobiota tends to demonstrate seasonal patterns and contribute to the degradation of polysaccharides and carbon cycling (Cardman et al. 2014; Orellana et al. 2022). This can imply their involvement in the consumption and degradation of complex algal polysaccharides, especially during diatom blooms (Orellana et al. 2022). The appearance of Verrucomicrobiota, especially in the year 2020 (Figure 6), although not dominant, can be attributed to the diatom bloom. Additionally, in the year 2019, during the late increase in diatom abundance in July (Supplementary Figure S7), it was possible to observe an increase in the relative abundance of Verrucomicrobiota.

One limitation of our study is the variability in filtered water volumes, which ranged from 1.2–5 L. This variation was primarily caused by filter clogging driven by differences in phytoplankton biomass and glacier‐derived sediment concentrations. Although Pascoal et al. (2023) demonstrated that filtered volume has minimal impact on microbial diversity estimates, uneven filtration volumes may still influence biomass capture and community representation. Moreover, our sampling schedule was constrained by local weather conditions, producing irregular temporal spacing between 2019 and 2020. Only samples that passed strict sequencing‐quality thresholds were retained, which further reduced sample counts, particularly for bottom waters in 2019, and may introduce bias in richness and diversity estimates.

### 4.2 Interactions Between Prokaryotic Communities

The network analysis performed in this study demonstrated several complex relationships between the communities, reflecting strong connections between the different network modules identified. This suggests that the microbial communities in Kongsfjorden are relatively stable in terms of structure (Röttjers and Faust 2018). Indeed, the networks analysed in this study were highly interconnected, as observed by Liu et al. (2023), except for the 2020 surface water prokaryotic communities. This less dense network can be due to the dominance of cold and local water masses in the spring and summer of 2020 (Supplementary Figure S2). A reduction in Atlantic inflow and horizontal dispersal limited community connectivity, resulting in increased compartmentalization and higher modularity (Table 2) (Watts and Strogatz 1998; Liu et al. 2023). These colder conditions likely slow the prokaryotes' metabolic rates and reduce primary production, thus limiting mutualistic interactions represented by positive co‐occurrence edges (Teeling et al. 2012; Sunagawa et al. 2015). The notable reduction of autotrophic prokaryotes in the spring and summer of 2020, such as Cyanobacteria (Figure 6; Supplementary Figure S6), likely diminished the flow of autotroph‐derived energy within the community (Falkowski et al. 2008), removing central taxa that typically act as network hubs (Teeling et al. 2012). This disruption possibly eliminated prokaryote–prokaryote interactions that rely on primary production, leading to a less connected network. Previous studies (e.g., Sogin et al. 2006; Pedrs‐Ali 2012; Lynch and Neufeld 2015) demonstrated that low‐abundance taxa have the capacity to act as “gatekeepers” of the communities that are present. Surface 2020 is mainly Bacteroidota and Proteobacteria (Figure 6), meaning that compositional dominance by a few taxa could explain the reduced connectivity. Additionally, functional reorganisation, like metabolic capabilities, transcriptional states, viral predation, or DOM transformation, can reshape interaction networks without major taxonomic change, creating faint co‐occurrence patterns undetectable by 16S amplicon methods (Watts and Strogatz 1998; Sunagawa et al. 2015).

The majority of interactions in this study were positive (58%–69%), suggesting that interactions were mainly cooperative between the prokaryotes (Zhang et al. 2022). Microbial interactions are influenced by both abiotic factors, such as pH and temperature, and biotic factors, like competition and predation (Steele et al. 2011; Sun et al. 2021). While many environmental parameters did not significantly change across the spring and summer of 2019 and 2020, there were notable differences in nitrate and phosphate concentration and temperature range at the surface (Figure 2a,e,f). Zhang et al. (2022) observed an increase in the number of interconnections as temperatures decreased with the onset of the cold season, but the lowest temperature was recorded at the surface in 2020, which, in our study, exhibited the lowest number of network edges. Higher diversity and a greater number of vertices in the networks generally indicate that increased biodiversity may promote more interactions within microbial communities (Czárán et al. 2002). The surface of 2020 exhibited the lowest diversity (Figure 5), which can serve as a hypothesis for the small network observed at the surface that year.

The network analysis in our study also revealed that the surface waters of 2020 exhibited high modularity (Modularity = 0.90). High modularity indicates a well‐organised structure where microbial communities are highly cohesive within their respective modules but exhibit few interactions with other modules (Mo et al. 2021; Liu et al. 2022). This pattern aligns with microbial communities in oligotrophic environments, where resources are limited, and microorganisms tend to form specialised, functional groups (Liu et al. 2022). This high modularity observed in the 2020 surface network suggests that microbial communities were adapted to local environmental conditions, forming distinct, ecologically specialised groups. These modules likely represent niche‐differentiated populations that are well‐adapted to the specific temperature, nutrient, and physicochemical conditions in the surface waters of 2020. The low interaction frequency between these modules indicates that, although these groups were well‐established, they did not extensively interact with other groups, possibly due to ecological specialisation or competition for limited resources (Faust and Raes 2012). Alternatively, it might reflect the presence of transient communities originating from different sources, such as glacier plumes. One of the key genera found exclusively in the surface network of 2020, *Pseudohongiella* (Proteobacteria), has been previously described as a typical component of bacterial communities linked to terrestrial runoff entering marine environments (Wietz et al. 2024). Wieber et al. (2025) demonstrated that terrestrial runoff, including glacial melt, is a significant source of biological material to Arctic coastal systems, including microbial taxa and ice‐nucleating particles, and plays a key role in shaping microbial community composition during periods of increased freshwater input.

*Octadecabacter* (Proteobacteria) was found exclusively in the surface network of 2020, a psychrophilic genus commonly associated with cold environments, including sea ice (Vollmers et al. 2013). Given that this genus only showed significant interactions on the surface of 2020, it may be persistent in sea ice or seasonal transitions involving sea ice melting. In addition, the genus *Psychromonas* (Proteobacteria) only demonstrated significant interactions at the bottom of 2019. This genus is also known for its adaptation to cold environments and for being present in deeper, nutrient‐rich water layers (Ramasamy et al. 2023). In this study, the concentration of nitrates and phosphate (Figure 2e,f) was elevated. The significant differences in the network between 2019 and 2020 and the two different depths might reflect the changing physical oceanographic conditions, like changes in ice cover and the influence of different glacier plume dynamics. This is very visible if we compare the network of surfaces of 2019, a more Atlantic environment, with the network of surfaces of 2020, a more Arctic environment.

## 5 Conclusion

This study provides new insights into the dynamics of prokaryotic communities in Kongsfjorden, highlighting their response to environmental changes over two contrasting years. Water masses, seasonal bloom dynamics, and associated physicochemical changes emerged as the primary factors shaping prokaryotic community composition and diversity. The differences in microbial networks between 2019 and 2020 may reflect the influence of varying water masses, with Atlantic water intrusion potentially contributing to the patterns observed in 2019, and local Arctic waters in 2020 supporting a different community structure. Temporal variability was evident, with the spring bloom dynamics significantly impacting prokaryotic diversity and community composition. Bacteroidota and Proteobacteria were the dominant phyla, underlining their pivotal role in organic matter degradation and nutrient cycling, while Crenarchaeota and Nitrospinota may contribute to nitrogen availability dynamics. Temporal shifts in prokaryotic communities were closely tied to the progression of the phytoplankton spring bloom, with phyla such as Verrucomicrobiota, Crenarchaeota, and Marinimicrobia (SAR406 clade) responding to changes in organic matter and nutrient availability. These findings underscore the critical role of microbial communities in Arctic ecosystems, particularly in mediating nutrient cycles under changing environmental conditions. Even though the study was limited to spring and summer and filtered volumes, as well as uneven sample representation across depths and years, our analysis suggests robust ecological patterns. The contrasting patterns between 2019 and 2020 underline the impact of Atlantification, which introduces taxa common to Atlantic waters or alters local microbial dynamics, ultimately reshaping ecosystem functions. This study enriches the understanding of microbial ecology in polar environments, reinforcing the importance of long‐term monitoring to track ecological changes and develop strategies to mitigate climate impacts on Arctic ecosystems. It underscores prokaryotic communities as indicators of and contributors to Arctic transformations, offering crucial insights into their roles in maintaining ecosystem stability under changing environmental conditions.

## Author Contributions

**Eva Lopes:** conceptualization, methodology, investigation, visualisation, writing – original draft, writing – review and editing, formal analysis; **Miguel Semedo:** conceptualization, writing – original draft, writing – review and editing, visualisation, methodology, validation; **Allison Bailey:** investigation, methodology, funding acquisition, project administration, writing – review and editing; **Philipp Assmy:** methodology, investigation, writing – original draft, writing – review and editing, funding acquisition, project administration; **Catarina Magalhães:** conceptualization, writing – original draft, writing – review and editing, validation.

## Funding

The Portuguese Science and Technology Foundation (FCT) funded this study through the grants to CM (2022.02983.PTDC; COMPETE2030‐FEDER‐00873300‐17183; 2023.14123.PEX), a PhD grant to EL (2022.13574.BD) and a work contract to MS under the Scientific Employment Stimulus (2023.08554.CEECIND/CP2848/CT0004). This research was funded by national funds through FCT, I.P., and by the European Commission's Recovery and Resilience Facility, within the scope of UID/04423/2025, UID/PRR/04423/2025, and LA/P/0101/2020. PA was funded by the Centre for Ice, Cryosphere, Carbon and Climate (iC3) supported by the Research Council of Norway through its Centres of Excellence funding scheme (grant no. 332635).

## Conflicts of Interest

The authors declare no conflicts of interest.

## Supporting information


**Figure S1:** emi470282‐sup‐0001‐FigureS1.tiff.

**Figure S2:** emi470282‐sup‐0002‐FigureS2.tiff.

**Figure S3:** emi470282‐sup‐0003‐FigureS3.tiff.

**Figure S4:** emi470282‐sup‐0004‐FigureS4.tiff.

**Figure S5:** emi470282‐sup‐0005‐FigureS5.tiff.

**Figure S6:** emi470282‐sup‐0006‐FigureS6.tiff.

**Figure S7:** emi470282‐sup‐0007‐FigureS7.tiff.

**Table S1:** emi470282‐sup‐0008‐TableS1.pdf.

**Table S2:** emi470282‐sup‐0009‐TableS2.pdf.

**Table S3:** emi470282‐sup‐0010‐TableS3.pdf.

**Table S4:** emi470282‐sup‐0011‐TableS4.pdf.

**Table S5:** emi470282‐sup‐0012‐TableS5.pdf.

## Data Availability

The environmental data that support the findings of this study are openly available in Norwegian Polar Data Centre at https://data.npolar.no/dataset/3c07e5ad‐c306‐4465‐973a‐d5d78b6769be, reference number https://doi.org/10.21334/NPOLAR.2024.3C07E5AD. The genomics data that support the findings of this study are available in the European Bioinformatics Institute (ENA) under the project PRJEB105035.
