# Supplementary figures and images for: Differences in Prokaryotic Community Composition Between Two Climatically Contrasting Years in an Arctic Fjord Ecosystem
Source: Environ Microbiol Rep. 2026 Apr 1;18(2):e70282. doi: 10.1111/1758-2229.70282 (PMC13045347; doi:10.1111/1758-2229.70282)

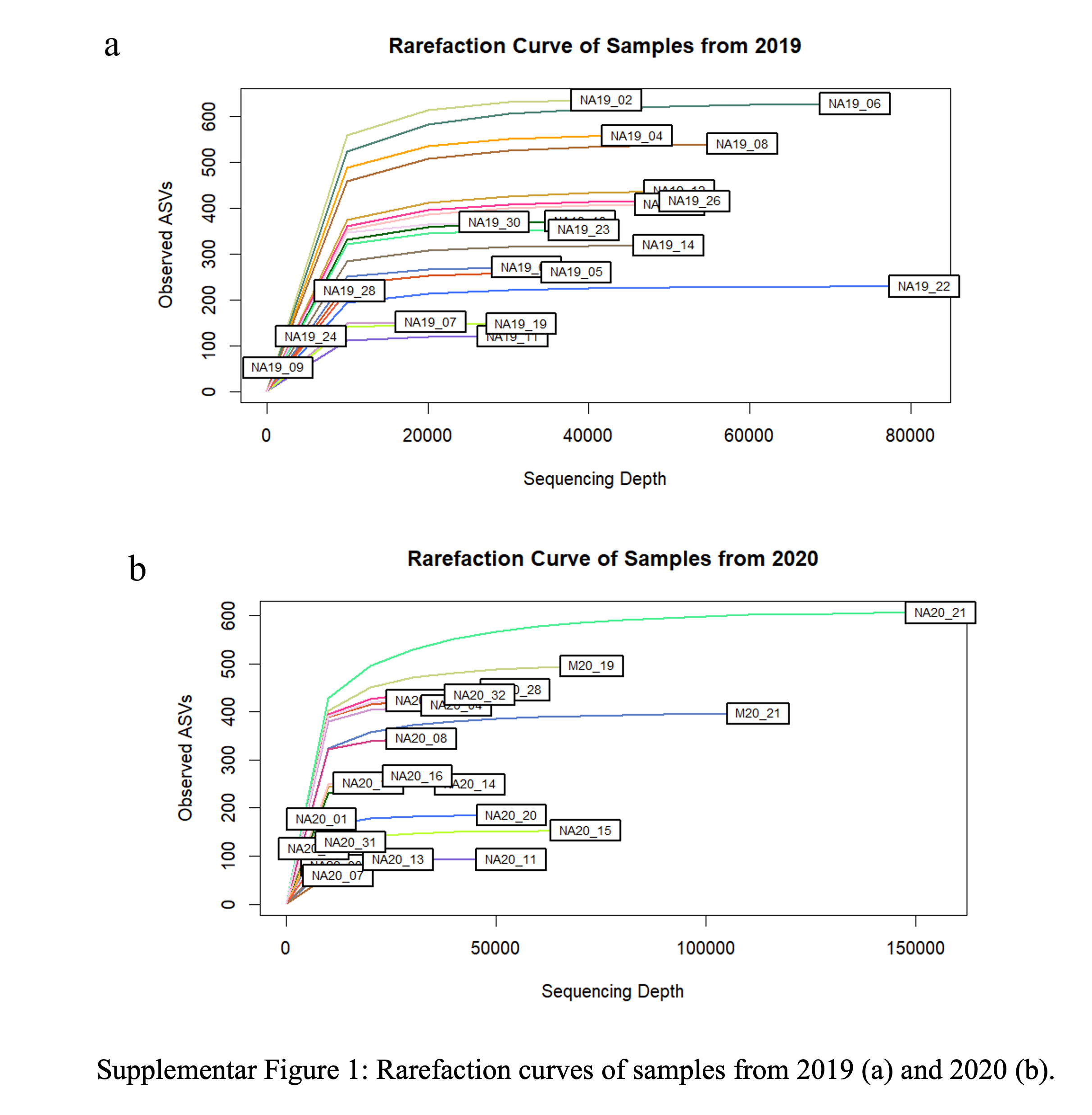

Supplement: Supplementary file 1 — Figure S1: emi470282‐sup‐0001‐FigureS1.tiff. [file EMI4-18-e70282-s008.tiff]

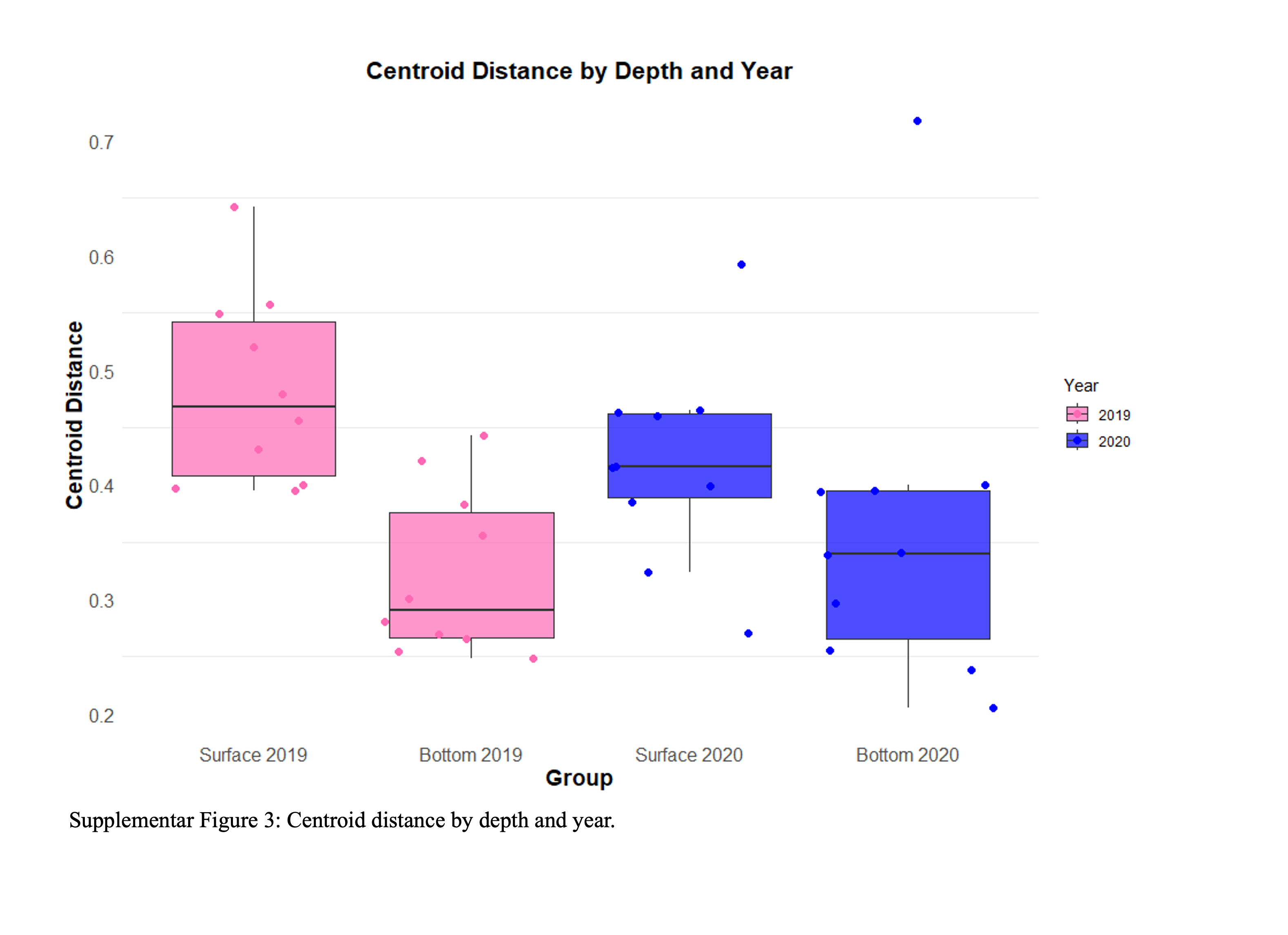

Supplement: Supplementary file 3 — Figure S3: emi470282‐sup‐0003‐FigureS3.tiff. [file EMI4-18-e70282-s011.tiff]

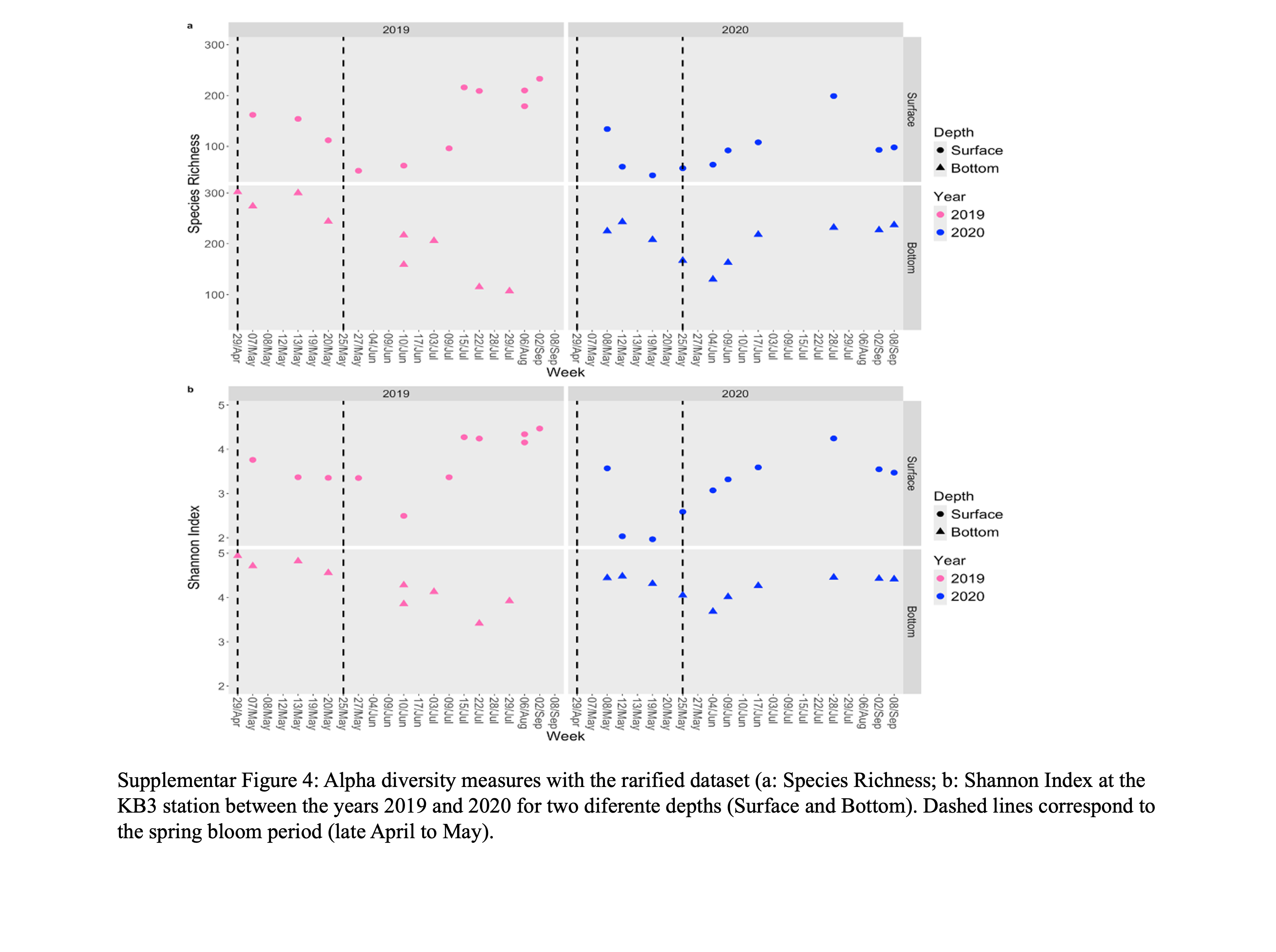

Supplement: Supplementary file 4 — Figure S4: emi470282‐sup‐0004‐FigureS4.tiff. [file EMI4-18-e70282-s006.tiff]

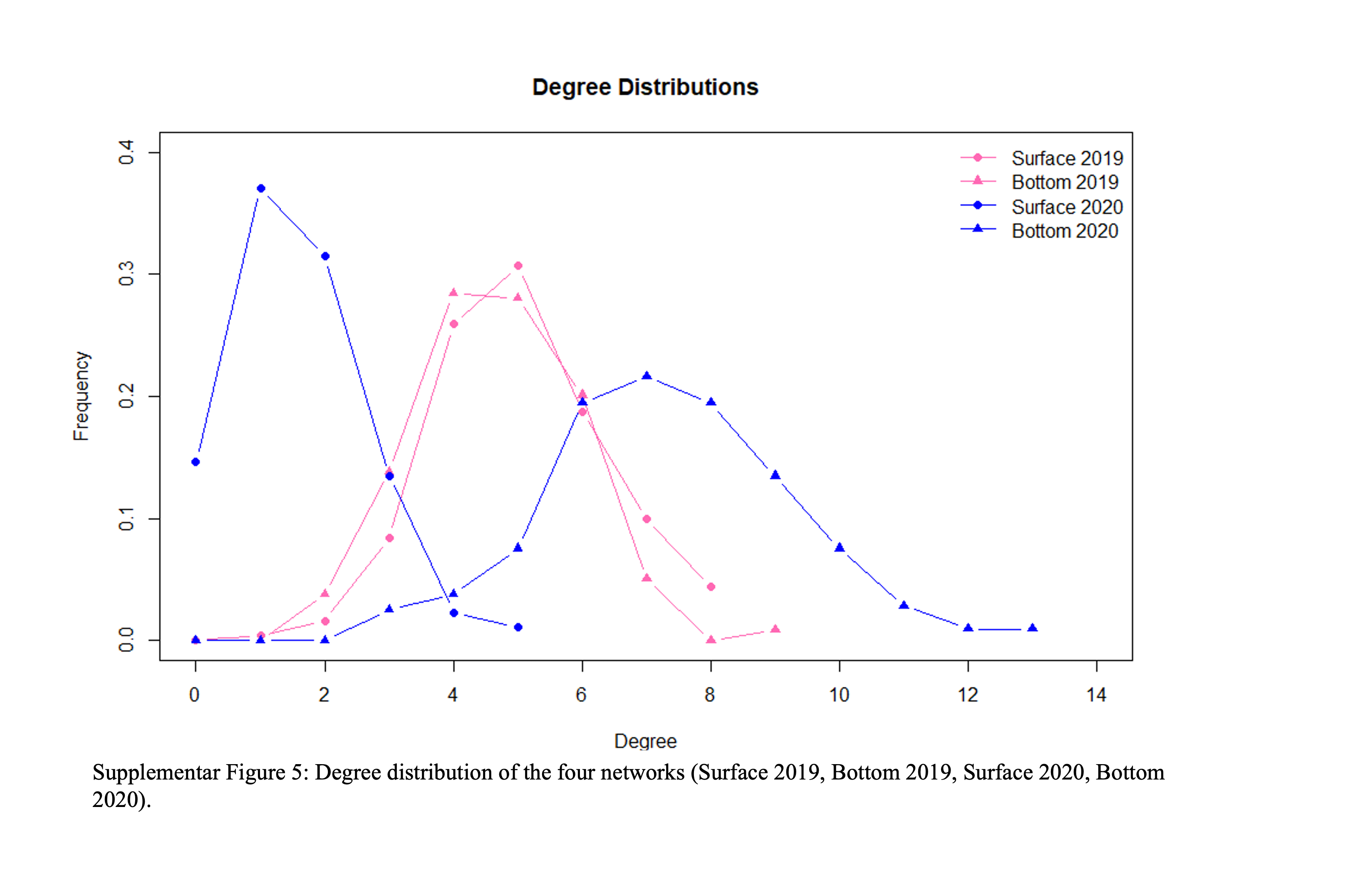

Supplement: Supplementary file 5 — Figure S5: emi470282‐sup‐0005‐FigureS5.tiff. [file EMI4-18-e70282-s012.tiff]

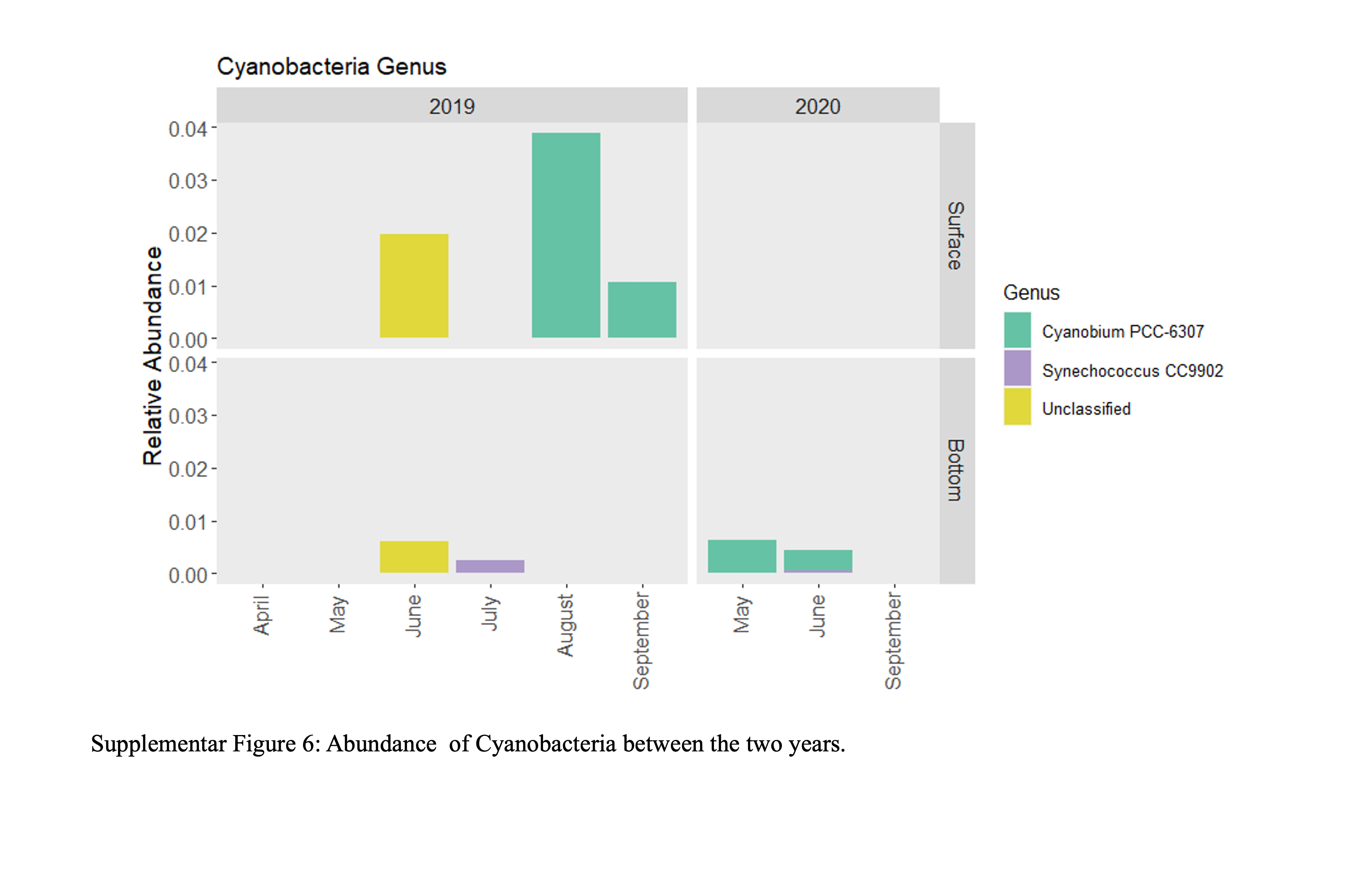

Supplement: Supplementary file 6 — Figure S6: emi470282‐sup‐0006‐FigureS6.tiff. [file EMI4-18-e70282-s002.tiff]
