# Supplementary material for: Differences in Prokaryotic Community Composition Between Two Climatically Contrasting Years in an Arctic Fjord Ecosystem
Source: Environ Microbiol Rep. 2026 Apr 1;18(2):e70282. doi: 10.1111/1758-2229.70282 (PMC13045347; doi:10.1111/1758-2229.70282)
Supplement: Supplementary file 8 — Table S1: emi470282‐sup‐0008‐TableS1.pdf. [file EMI4-18-e70282-s004.pdf]

Supplementary Table 1: Steps of the upstream analysis.

Year	Date	Sample ID	Depth (m)	Input	Filtered	DenoisedF	DenoisedR	Merged	Nonchim	Final Output*
2019	29 April	NA19_02	300	52434	47090	46369	46546	45026	44716	42207
	07 May	NA19_03	15	77953	70488	69873	70033	67079	66663	32303
		NA19_04	300	59735	53069	52490	52648	50962	50345	46002
	13 May	NA19_05	15	82499	74847	74584	74611	71114	70850	38456
		NA19_06	300	91738	82118	81142	81430	78205	77069	73134
	20 May	NA19_07	15	35166	31954	31834	31859	31241	31098	20381
		NA19_08	300	74199	66518	65596	65914	62904	62032	59139
	27 May	NA19_09	15	2495	2255	2208	2197	2102	2087	1383
	10 June	NA19_11	15	37826	34423	34199	34226	33587	33567	30603
		NA19_12	300	63666	56581	55859	56058	53968	53303	51253
	20 June	NA19_14	300	64296	57959	57557	57691	56128	55943	49909
	03 July	NA19_18	300	46911	42393	41790	41997	40016	39788	38935
	09 July	NA19_19	15	45842	41508	41103	41234	40086	39902	31612
	15 July	NA19_21	15	58638	52949	52281	52510	50999	50739	50100
		NA19_22	300	98466	88671	88256	88330	86714	85095	81725
	22 July	NA19_23	15	46148	41542	40894	41204	39908	39761	39432
		NA19_24	300	7357	6535	6425	6405	5995	5951	5445
	06 August	NA19_26	15	60824	55204	54577	54821	53774	53540	53217
	26 August	NA19_28	15	12535	11140	10912	10932	10396	10377	10302
	02 September	NA19_30	15	33633	30140	29549	29836	28581	28476	28271
2020	28 July	M20_19	339	99916	89506	87615	88414	79734	73320	72833
		M20_21	25	188380	169046	166273	167252	149406	119148	112483
	08 May	NA20_01	15	15397	13920	13719	13777	13269	13239	8434
		NA20_02	300	42116	37717	37218	37306	35600	35350	32229
	12 May	NA20_03	15	15179	13709	13519	13589	13276	13238	12013
		NA20_04	300	53267	47123	46419	46715	45364	45032	40530
	19 May	NA20_07	15	16386	14588	14533	14557	14182	14178	12428
		NA20_08	300	40397	36301	35769	35941	34852	34596	32232
	25 May	NA20_11	15	62903	57090	56927	56981	56604	56203	53623
		NA20_12	300	23736	21449	21059	21220	20419	20343	19592
	04 June	NA20_13	15	30639	27704	27548	27626	27354	27345	26723
		NA20_14	300	53325	47791	47425	47545	45829	45680	43745
	09 June	NA20_15	15	81068	72819	72690	72684	72290	72225	71304
		NA20_16	300	40532	36535	36179	36290	34028	33948	31277
	17 June	NA20_20	15	61327	55701	55395	55500	54745	54603	53701
		NA20_21	300	186799	167264	166399	166751	162112	161356	155907
	02 September	NA20_27	15	7885	7072	6986	6973	6815	6736	6575
	08 September	NA20_28	300	64809	57528	56787	56997	55297	54803	54585
		NA20_31	15	18864	17055	16832	16891	16352	16195	15384
		NA20_32	300	55033	49384	48493	48886	46840	46294	46145

*: Non-chimeric reads that remain after ASVs classified as eukaryotic, mitochondrial, and chloroplast were removed.
