# Supplementary material for: Differences in Prokaryotic Community Composition Between Two Climatically Contrasting Years in an Arctic Fjord Ecosystem
Source: Environ Microbiol Rep. 2026 Apr 1;18(2):e70282. doi: 10.1111/1758-2229.70282 (PMC13045347; doi:10.1111/1758-2229.70282)
Supplement: Supplementary file 9 — Table S2: emi470282‐sup‐0009‐TableS2.pdf. [file EMI4-18-e70282-s007.pdf]

Supplementary Table 2: Permutational multivariate analysis of variance (PERMANOVA) output.

Factor	DF	R2	p-value
Year	1	0.23765	0.001
Depth	1	0.07727	0.002
Year*Depth	1	0.07076	0.001
Residuals	36	0.61432	-
Year Beta Dispersion	1	-	0.395
Depth Beta Dispersion	1	-	0.008
