# Supplementary material for: Differences in Prokaryotic Community Composition Between Two Climatically Contrasting Years in an Arctic Fjord Ecosystem
Source: Environ Microbiol Rep. 2026 Apr 1;18(2):e70282. doi: 10.1111/1758-2229.70282 (PMC13045347; doi:10.1111/1758-2229.70282)
Supplement: Supplementary file 10 — Table S3: emi470282‐sup‐0010‐TableS3.pdf. [file EMI4-18-e70282-s005.pdf]

Supplementary Table 3: Envfit output for the surface and bottom of 2019 and 2020.

		**R2**	**p-value**
Surface	Salinity	0.3543	0.100899
	Temperature	0.3994	0.089910
	Fluorescence	0.0374	0.858142
	Turbidity	0.0419	0.761239
	Nitrate	0.5856	0.005994
	Nitrite	0.2413	0.279720
	Phosphate	0.4853	0.022977
	Silicic Acid	0.7282	0.000999
	Ammonium	0.4808	0.061938
	Chlorophyll-a	0.0202	0.929071
	Phaeopigments	0.0182	0.936064
	Diatoms	0.1163	0.384615
	Phaeocystis	0.0378	0.805195
Bottom	Salinity	0.0512	0.582418
	Temperature	0.5202	0.041958
	Fluorescence	0.6385	0.006993
	Turbidity	0.2676	0.142857
	Nitrate	0.6381	0.003996
	Nitrite	0.0399	0.732268
	Phosphate	0.6938	0.001998
	Silicic Acid	0.4670	0.029970
	Ammonium	0.1153	0.250749
