# Supplementary material for: Differences in Prokaryotic Community Composition Between Two Climatically Contrasting Years in an Arctic Fjord Ecosystem
Source: Environ Microbiol Rep. 2026 Apr 1;18(2):e70282. doi: 10.1111/1758-2229.70282 (PMC13045347; doi:10.1111/1758-2229.70282)
Supplement: Supplementary file 11 — Table S4: emi470282‐sup‐0011‐TableS4.pdf. [file EMI4-18-e70282-s009.pdf]

Supplementary Table 4: Linear regression output of bloom indicators.

	Species Richness				Species Diversity			
	Slope	Y-Intercept	R2	p-value	Slope	Y-Intercept	R2	p-value
Fluorescence	-59.91	349.17	0.2364	0.001968	-0.30428	4.17567	0.2852	0.000554
Chlorophyll-*a*	-29.65	235.97	0.2313	0.0433	-0.21121	3.7962	0.3315	0.0124
