# Supplementary material for: Differences in Prokaryotic Community Composition Between Two Climatically Contrasting Years in an Arctic Fjord Ecosystem
Source: Environ Microbiol Rep. 2026 Apr 1;18(2):e70282. doi: 10.1111/1758-2229.70282 (PMC13045347; doi:10.1111/1758-2229.70282)
Supplement: Supplementary file 12 — Table S5: emi470282‐sup‐0012‐TableS5.pdf. [file EMI4-18-e70282-s003.pdf]

Supplementary Table 5: Relative abundance of the phyla between the two years at the different depths.

	Surface 2019	Bottom 2019	Surface 2020	Bottom 2020
Acidobacteriota	0.000445756	0.003326004	0.000702422	0.002121372
Actinobacteriota	0.020960819	0.037883232	0.011268453	0.043185492
Altiarchaeota	0	5.13677E-05	0	0.000102083
AncK6	7.80112E-05	0.000318448	0	0
Asgardarchaeota	0	4.73855E-05	0.000456274	0
Bacteroidota	5.263226395	3.35441927	6.624589199	3.931233032
Bdellovibrionota	0.020100421	0.012693536	0.003216524	0.019063122
Campylobacterota	3.99202E-05	5.46941E-05	0	0.000223623
Chloroflexi	0.002849287	0.021222546	0.001811823	0.019562221
Crenarchaeota	0.507139699	0.347784166	0.202118633	0.152078368
Cyanobacteria	0.000690449	8.45817E-05	0	1.28282E-05
Dadabacteria	0.031960419	0.110374338	0.022189421	0.041892292
Deinococcota	0	0.002925132	0	0.000645298
Dependentiae	0.000388274	2.73471E-05	0	2.74601E-05
Desulfobacterota	0.002751175	0.003670418	0.004165533	0.004908827
Elusimicrobiota	0.001593017	5.13677E-05	0	1.28282E-05
Entotheonellaeota	0	6.52146E-05	0	8.66833E-05
Fibrobacterota	0.000797033	0.002442784	2.66707E-05	0.00329213
Firmicutes	0.013014797	0.01224221	0.002224004	0.002583559
Fusobacteriota	0.000291206	0.00016194	0.000355703	9.30839E-05
Gemmatimonadota	0.006277538	0.029993003	0.015733907	0.022804425
Halobacterota	0	0.000377252	0	0
Hydrogenedentes	0.000290048	0.000924376	0	0.00066382
Latescibacterota	5.98802E-05	9.90332E-05	0	0.000122488
Margulisbacteria	0.000318347	0.000698087	0	0.00097226
Marinimicrobia (SAR406 clade)	0.05334215	0.128439257	0.036674115	0.135422293
Myxococcota	0.00308978	0.003167233	0.001009183	0.002778355
Nanoarchaeota	0.005591544	0.004281858	0.000744189	0.005159117
NB1-j	0.025951228	0.021007214	0.017798846	0.035793676
Nitrospinota	0.385882292	0.112318705	0.281602742	0.194749155
Nitrospirota	5.07202E-05	9.4771E-05	0	3.20704E-05
NKB15	0	4.73855E-05	0	5.49602E-05
PAUC34f	0.000508222	0.006403241	0.000593485	0.004878764
Planctomycetota	0.29204622	0.217951352	0.018264024	0.390426671
Proteobacteria	3.936129992	3.861368746	1.933575361	4.577307943
SAR324 clade(Marine group B)	0.072308828	0.147694252	0.105270869	0.159868966
Spirochaetota	0.000804286	3.90221E-05	0.00014956	0.000706926
Sumerlaeota	0	0	0	5.49602E-05
Sva0485	0	9.4771E-05	0	0
Thermoplasmatota	0.210114171	0.478258782	0.546610573	0.187568308
Unclassified	0.00477196	0.002122166	0.000330621	0.002889911
Verrucomicrobiota	0.136136119	0.072901784	0.168405592	0.05530237
WPS-2	0	0.001871727	0	0.00131836
